# Metabolite Profiling in the Pursuit of Biomarkers for IVF Outcome: The Case for Metabolomics Studies

**DOI:** 10.1155/2013/603167

**Published:** 2013-01-16

**Authors:** C. McRae, V. Sharma, J. Fisher

**Affiliations:** ^1^School of Chemistry, University of Leeds, Leeds LS2 9JT, UK; ^2^The Leeds Centre for Reproductive Medicine, Leeds Teaching Hospitals NHS Trust, Seacroft Hospital, Leeds LS14 6UH, UK

## Abstract

*Background*. This paper presents the literature on biomarkers of *in vitro* fertilisation (IVF) outcome, demonstrating the progression of these studies towards metabolite profiling, specifically metabolomics. The need for more, and improved, metabolomics studies in the field of assisted conception is discussed. *Methods*. Searches were performed on ISI Web of Knowledge SM for literature associated with biomarkers of oocyte and embryo quality, and biomarkers of IVF outcome in embryo culture medium, follicular fluid (FF), and blood plasma in female mammals. *Results*. Metabolomics in the field of female reproduction is still in its infancy. Metabolomics investigations of embryo culture medium for embryo selection have been the most common, but only within the last five years. Only in 2012 has the first metabolomics investigation of FF for biomarkers of oocyte quality been reported. The only metabolomics studies of human blood plasma in this context have been aimed at identifying women with polycystic ovary syndrome (PCOS). *Conclusions*. Metabolomics is becoming more established in the field of assisted conception, but the studies performed so far have been preliminary and not all potential applications have yet been explored. With further improved metabolomics studies, the possibility of identifying a method for predicting IVF outcome may become a reality.

## 1. Introduction

Infertility is an extremely prevalent problem, affecting one in every seven couples [1, 2], and as result *in vitro* fertilisation (IVF) has become increasingly popular since it was pioneered in 1978. In 2010, 45 264 women were treated by IVF in the UK [3], whereas in 1992, 14 057 women were treated [4]. As understanding of fertility and embryology has developed, the procedures and techniques in assisted conception have been ever improving, and as a result, the number of live births resulting from IVF or ICSI has increased since the early 90s [3]. The UK live birth rate per IVF cycle in 1991 was 14.0% and this increased to 24.1% by 2009 [3]. In 2009 the live birth rate per ART treatment in Canada was higher, at 27.4% [5] and higher still in the USA at 31% [6] but lower in Australia and New Zealand at 17.2% [7]. While improved, these success rates are still unsatisfactorily low. The development of controlled ovarian stimulation in the 1980s [8] enabled the production of multiple mature oocytes and hence multiple embryo transfer, improving the chances of pregnancy in an IVF cycle. Today, gonadotrophins are routinely administered to women undergoing IVF in an attempt to improve the chances of conception. The drawback to multiple embryo transfer, however, is that the patient has a greater chance of developing a multiple pregnancy, which carries an increased risk of maternal and infant morbidity. Infants from multiple pregnancies are more likely to suffer late midtrimester miscarriage or have a low birth weight and/or be born prematurely and as a result may require intensive neonatal care facilities and are at greater risk of being born with long term disabilities such as cerebral palsy, deafness, and visual impairment requiring long term support services [9]. The most common maternal complications associated with multiple pregnancies include high blood pressure, preeclampsia, increased likelihood of caesarean section, venous thromboembolism, postpartum haemorrhage, and gestational diabetes [10]. Consequently, multiple births are far more costly to the National Health Service (NHS) than singleton births [11]. A study from 2009 estimated the total cost of preterm births from birth to adult life, in the public sector in England and Wales, to be *£*2.946 billion [12]. This study also reported that 92% of this cost per preterm survivor was due to hospital inpatient services [12]. From 2004, the Human Fertilisation and Embryology Authority (HFEA) policy was to transfer no more than two embryos to women under the age of 40, and no more than three into woman aged 40 or over [13]. Although this resulted in a decrease in rates of triplet births, the occurrence of twins remained high and in 2008, when nearly 24% of all IVF births resulted in multiple pregnancies, the HFEA introduced a policy to bring down the UK IVF multiple birth rate to 10% over a period of several years [14]. To achieve this goal, the HFEA has promoted the transfer of just one embryo for women who have a high chance of becoming pregnant [14]. This is known as “elective single embryo transfer” (eSET) [14]. This policy has been adopted in other countries as well. The latest figures from the HFEA showed that in 2010 the majority of embryo transfers in the UK were double (64.8%) and that just 14.9% were eSET [3], though this is an improvement from 2008 when only 4.8% of embryo transfers performed were eSET. Just 4.4% were triple embryo transfers to women over 40 (the remaining percentage of transfers were single, but because only one embryo was available, these were not elective) [3]. In Canada, in 2009, 13% of embryo transfers were eSET and the majority were double (58%) or triple (22%) [5]. In the USA in the same year, rates of eSET were 7.4% for women below 35 years, 2.8% for women between 35 and 40 years, and 0.5% among women older than 40 years [6]. The latest figures from Europe, in 2008, reported 22.4% of embryo transfers were single, 53.2% were double, and 24.4% were triple or more [15]. However, the highest proportion of eSET is being performed in Australia and New Zealand; in 2009, 69.7% of embryo transfers were single [7], reflecting not only the lower multiple birth rate, but also the lower (live) birth rate relative to the other countries. The multiple pregnancy rate in 2010 in the UK was 21.8% [3]. In Canada, the USA, and Australia and New Zealand in 2009, the multiple pregnancy rates were 27.4% [5], 47.3% [6], and 8.2% [7], respectively. In 2008, the multiple pregnancy rate in Europe was 21.7% [15]. Thus, while multiple embryo transfers remain high, so too is the risk of multiple pregnancies. There are many factors that dictate the selection of embryo/s for transfer; in the UK this includes limited NHS funding in most areas. It is important to have more confidence in performing an eSET to ensure that the patient's chances of success are not compromised. 

While IVF is hampered by poor success rates and the increasing pressure to reduce multiple embryo transfers, a major objective in reproductive medicine currently is to find a method for identifying the best embryos for transfer. This will allow for eSET without a compromise in success rates. Furthermore, considering the emotional, health, and financial commitments involved in fertility treatment, finding a reliable method for embryo selection is clearly desirable. Therefore a great body of research has been undertaken with aims of identifying biomarkers of oocyte and embryo quality, the majority of which have been targeted analyses; where one class of biological compound is measured and examined for its predictive ability. Such studies are still being performed, but as of yet no definitive biomarker to predict IVF outcome has been identified. Therefore, studies are required where many classes of compounds can be investigated for predictive ability simultaneously, not only to increase the chance of identifying a marker but also because if single markers cannot be found, it may be that only *combinations* of biomolecules provide a diagnostic. Furthermore, in reality, a group of biomarkers would be of more clinical value than one alone, since a simultaneous change of several species together adds confidence and validity to the diagnosis and is less likely to be incidental, as might be the case for a rise or fall in levels of a single metabolite. With these points in mind biomarker research in the field of assisted conception is moving in the direction of global metabolite profiling, or metabolomics.

Metabolomics (akin to metabonomics [16]) is the non-targeted identification and quantification of all the metabolites in the metabolome [17, 18], where metabolites are the low molecular weight end products and starting materials of essential cellular reactions, and the metabolome is the complete collection of all the metabolites in an organism [19]. Biofluids, such as blood plasma, perfuse, and are in dynamic equilibrium with cells, therefore by measuring which metabolites are present in the biofluid and the levels of these metabolites (i.e., the metabolic profile of the biofluid) it may be possible to obtain information about the metabolism within those cells [16]. The presences of metabolites in a biofluid are often indicated by identifying the functional groups that exist within the molecular structure of the metabolites, for example, carboxylic acids, ketones, aldehydes, alkenes, or alkynes. Ultimately it may be possible to discriminate between biofluids from different groups of individuals, for example, diseased versus controls, based on their metabolic profiles. If it was possible to discriminate between such groups, it may also become possible to classify other, unknown, samples based on their metabolic profiles, and hence identify biomarkers for certain diseases. Metabolomics can be applied to assisted conception by obtaining and examining the metabolic profiles of biofluids associated with oocytes (follicular fluid (FF)) and embryos (culture medium or blastocoele fluid) for biomarkers of oocyte/embryo quality. One of the most common analytical platforms for metabolomics is nuclear magnetic resonance (NMR) spectroscopy, which measures the presence of certain nuclei (most commonly, ^1^H) in a sample, producing a spectrum, where the positions of the signals on the *x*-axis (chemical shift in ppm) reflect the chemical environments of the corresponding nuclei (and hence enable identification of the metabolites), and the intensities of the signals indicate the number of nuclei in the given chemical environment (so the area under the signal gives a measure of the concentration of the metabolite). The second most commonly used platform is mass spectrometry (MS), which offers a much greater sensitivity than NMR (picomolar-femtomolar level [20] versus micromolar level in NMR [21]). However, NMR has several advantages over MS, including minimal sample preparation, rapid analysis time, and better reproducibility and it is nondestructive to the sample. MS requires that different classes of compound are analysed separately, and therefore is often coupled to a prechromatography step, which slows down analysis time and introduces reproducibility issues. Biofluid samples are analysed using these platforms (and others, e.g., infrared (IR) spectroscopy [22]) to produce data matrices containing measures of the levels of all the metabolites (columns) in each sample (rows), which are then interrogated using multivariate statistical analyses to identify trends in different groups of samples and the metabolites responsible for those trends. In this way, a biomarker or groups of biomarkers may be identified, and the class memberships of new samples predicted using these models.

A number of reviews have appeared over the last seven years highlighting the potential application of metabolomics in female reproduction. These include reviews specific to metabolomics of FF [23], embryo culture medium [24–29], oocytes and their related cells [28–30], and the introduction of metabolomics techniques to gynaecologists [31, 32]. The use of NMR spectroscopy alone in understanding the female reproductive tract, including studies of cervical mucus, uterine matter, the pelvis, ovarian tissue, and FF has also been reviewed [33]. The use of ^31^P-NMR in studying female reproduction has also been demonstrated [34, 35]. The aim of this paper is to review the progress so far in pursuit of biomarkers for IVF outcome and to highlight the recent progression of this research from targeted metabolite analysis to metabolomics.

## 2. Materials and Methods

Literature searches for publications written in the English language were performed using ISI Web of Knowledge. Key words used for the searches were “embryo quality,” “oocyte quality,” “embryo,” “oocyte,” “follicular fluid,” “blood plasma,” and “female fertility,” which were each paired with the following terms: “biomarker” (and in the case of “biomarker” paired with “embryo,” “oocyte,” “follicular fluid,” or “blood plasma”, an additional “IVF outcome” term was included in the search terms), “metabolomics,” and “metabonomics”. Animal as well as human studies were included, but papers focused on male subfertility/infertility were excluded, as they were studies on any species other than mammals (e.g., there were many studies on amphibian oocytes which were excluded). Studies on tissue or fluid postembryo transfer were not considered for this paper. Studies involving magnetic resonance imaging (MRI) and NMR microimaging were not considered. No restriction was set for the publication date.

## 3. Results and Discussion

### 3.1. Selecting the Best Embryo

Currently, embryos are selected for implantation based on assessments of their morphology and cleavage rates [27, 32]. However, while morphological assessments are quick and inexpensive, they are subjective and limited by a lack of standards and fail to identify genetic or epigenetic defects [32]. A further limitation of this “snapshot” assessment of embryos, which is that the dynamic nature of embryo development can affect its morphology score, has recently been overcome by the development of time-lapse monitoring of embryo development. Meseguer et al. [36] developed a hierarchical model for predicting embryo implantation potential based on various morphokinetic parameters measured by automatic image analyses of incubated human embryos. Most recently, the same group showed that the model was also able to predict embryos most likely to develop to the blastocyst stage [37]. While morphokinetics is becoming the new “gold standard” for determining embryo developmental potential, the methods are still in their infancy and limitations such as the influences of different culture media on the morphokinetic behaviour of embryos are still unknown [36]. Clinical markers for embryo quality may overcome some of the limitations of morphological assessments, such as subjectivity, and could be complementary to morphological or morphokinetic assessments. 

A large body of research has examined embryo metabolism as a predictor for embryo viability. These studies have analysed the uptake and secretion of various metabolites by the embryo into the surrounding culture medium. Early studies of animal embryos identified an elevated glucose uptake in good quality embryos compared to poorer quality ones [38, 39]. This observation has since been confirmed in human embryos [40, 41]. Houghton et al. [42] measured the turnover of amino acids by human embryos during culture, and observed that at all stages of embryo development examined, a low amino acid turnover was associated with better development. This finding was reinforced in a later study by the same group [43], along with the observation that decreased culture medium levels of glycine and leucine and increased levels of asparagine correlated with clinical pregnancy and live birth. Differences in amino acid turnover between genetically normal and abnormal embryos have also been observed during different stages of culture [44]. Embryo oxygen consumption has also been investigated as a method for assessing embryo quality, particularly in bovines; early studies showed a link between embryo oxygen consumption and embryo morphology [45, 46]. In 2005, Lopes and colleagues developed the Nanorespirometer [47], which offered a noninvasive and rapid method for measuring embryo respiration rates [48]. Using this method, however, Lopes et al. were unable to find a significant difference in respiration rates between bovine embryos which did and did not result in a pregnancy, though respiration rates increased with morphological quality *in vivo* [48]. Most recently, it has been suggested that monitoring patterns of oxygen consumption in human embryos in culture for up to 72 hours may be informative of embryo viability [49].

In recent years, studies of embryo metabolism have moved away from targeted metabolite analysis and towards metabolomics. The first metabolomics study of embryo culture medium to assess oocyte potential was carried out using near infrared (NIR) and Raman spectroscopy by Seli et al. in 2007 [50]. A multilinear regression algorithm was used to find the spectral regions that differed between embryos that implanted and led to a pregnancy and those which failed to implant, and viability scores calculated for each embryo [50]. Higher values of viability scores were associated with live birth [50]. The validity of Seli and colleagues' scoring system was tested in a blinded trial [51], in which Raman spectra of spent culture media from human embryos cultured at a different IVF centre and under some different conditions to those used by Seli et al. [50] were analysed. Again viability scores were significantly higher in embryos which implanted compared to those that did not [51]. Subsequently, larger scale studies have been conducted using similar analyses on NIR spectra from embryos undergoing transfer on various days of culture and have consistently shown higher viability scores for those that implanted and led to a pregnancy with foetal heart activity [52–54]. This algorithm generated using fresh embryos has also been shown to be predictive of viability of frozen-thawed embryos [55]. One study found the viability score to be more effective, either alone or in combination with morphologic assessment, than morphology alone in predicting embryo quality in women undergoing single embryo transfer on day 5 of culture [56]. More recently however, a single-centre randomized controlled trial showed no difference in pregnancy rate for single embryo transfers with embryo selection based on NIR analysis and morphological assessments compared to using morphology alone [57]. Marhuenda-Egea et al. [58, 59] have used high performance liquid chromatography (HPLC)-MS- and ^1^H-NMR-based metabolomics to identify differences in culture media from embryos with and without pregnancy. The group used soft independent modelling of class analogy (SIMCA) to classify samples as “pregnancy” or “nonpregnancy embryos” based on amino acid concentrations determined from the HPLC-MS analysis. The authors postulated that all embryo culture medium amino acids played a crucial role in the embryo metabolism [59]. For the NMR data, interval partial least squares (iPLS) analysis was used to identify the lipid region (0.5–1.2 ppm) as giving the best correlation with embryo pregnancy rate [59]. Recently a HPLC-MS-based metabolomic approach to examining blastocoele fluid (a fluid withdrawn from a blastocyst cavity prior to cryostorage) for markers of embryo quality has been described [60].

Although the studies described above have indicated that metabolic differences between embryos may be indicative of their potential to result in a pregnancy, the application of culture medium analysis has remained limited in the clinical setting [24]. In the cases of targeted metabolite studies, the technologies used tend to require costly equipment and technical expertise and do not produce results rapidly enough for embryos to be assessed in time for transfer [24]. Metabolomics also requires expensive equipment, such as spectrometers and chromatography systems, and specialist skills to perform the compositional and statistical analyses. However, because the analysis is global, metabolomics has the potential to identify several biomarkers simultaneously, making it more time and cost effective than performing several separate targeted analyses. Furthermore, the analysis of whole biofluids or tissue is more rapid because preseparation steps to isolate single metabolites are not required and the compositional analysis itself is usually high throughput. After initial capital outlay, individual tests are inexpensive, unlike assays. Ultimately, the goal of metabolomics is to generate a model, such as a scoring system, for example, in which new observations can be entered and their class memberships predicted. Therefore, eventually specialised knowledge may not be required, only the means of obtaining the compositional data. Furthermore, if a single or small number of biomarkers are identified, it may be possible to develop more simple, rapid, and affordable “pin prick” tests or assays that could be performed in an embryology laboratory. While metabolomics studies of embryo culture media are still relatively new and are still in the validation process, one reason why they have not yet taken off may be because they have been somewhat limited in terms of the range of culture media and days of embryo transfer investigated. In practice, IVF clinics use many types of culture media, use different volumes of media, and have variable policies/preferences regarding the optimum day for embryo transfer [28], and so it is uncertain whether the biomarkers and viability scores found thus far are generally applicable.

### 3.2. Selecting the Best Oocyte

An alternative approach to predicting the likelihood of pregnancy following IVF is to assess oocyte quality and to determine the best oocyte for fertilisation. Oocyte selection has the additional advantages of limiting embryo overproduction (which has ethical and storage implications) and improving the outcome of oocyte cryostorage programs [23]. Some methods for determining oocyte quality already exist, primarily involving studies of oocyte morphology; specifically of the cumulus-oocyte complex (CoC) [61, 62], cytoplasm [63], polar body [64–66], and/or meiotic spindle [67, 68], as well as the follicle itself [69–71]. These methods, however, tend to be more efficient for identifying oocytes of *poor* quality rather than good, and so while it is easy to rule certain oocytes out, it is more difficult to determine which is the best “good oocyte” [23]. Furthermore, the results of morphology studies have been very conflicting due to the large subjectivity and inaccuracy of these methods [72]. For similar reasons it has not been possible to investigate and/or compare treatment regimens for developing oocytes with optimum quality. An alternative line of research has produced studies aimed at finding cellular predictors of oocyte quality. Cellular predictors of *good* oocyte quality include a high content of oocyte mitochondrial DNA [73, 74]; adequate redistribution, differentiation, and transcription of mitochondria [75]; varying levels of adenosine triphosphate (ATP) in the cytoplasm produced by the oocyte [76]; and low glucose-6-phosphate dehydrogenase (G6PDH) activity in oocytes [77]. The problem with all of these methods, with the exception of the brilliant cresyl blue staining method used in [77], however, is that they are invasive and so do not allow preservation of the quality of the oocyte. Noninvasive targeted metabolite analysis of oocytes has been carried out, involving the measurement of energy substrate levels in culture media. Preis et al. [78] used ultramicrofluorimetry to quantify glucose and lactate consumption and release into culture media from mouse oocytes. They found the oocytes that consumed larger amounts of glucose and produced more lactate had the highest potential for fertilisation [78]. A large body of research on oocyte metabolism has been reported by a group at The University of Leeds. Key studies include an investigation of carbohydrate and oxygen consumption throughout murine oocyte development (*via* analysis of spent follicular culture media), which revealed higher rates of pyruvate and oxygen consumption during the primary follicle stage than the preovulatory and primordial stages [79]. A similar earlier study found significantly higher rates of glucose consumption and lactate and pyruvate production in *in vitro *grown murine CoCs than for *in vivo* ovulated controls [80]. The same group also showed that murine FF, oviductal, and uterine fluid compositions differ from those of murine maturation, fertilization, and embryo culture media, and suggested that this information could be used to create more physiologically accurate oocyte/embryo culture media [81]. Similarly, the carbohydrate and amino acid profile of bovine FF was measured to assess the physiological accuracy of bovine maturation medium [82]. It was thought that the nonphysiological nature of the culture media might be in part responsible for the poor developmental competence of bovine oocytes matured *in vitro* [82]. In another study, oocytes from patients suffering with polycystic ovary syndrome (PCOS) exhibited greater rates of glucose and pyruvate consumption compared to controls [83]. Higher pyruvate turnover was also associated with abnormal oocyte karyotypes [83]. Most recently this group have shown in bovines that oocyte amino acid turnover is predictive of oocyte blastocyst developmental competence using targeted profiling of *in vitro* maturation medium from oocytes prior to fertilisation [84].

Other studies have investigated the respiration rate of oocytes as a marker for oocyte viability; Scott et al. [85] found that human oocytes with respiration rates between 0.48 and 0.55 nL O_2_/h were viable, whereas those with lower rates did not mature or became atretic *in vitro*. More recently, Tejera et al. confirmed that oocytes with higher rates of oxygen consumption were those that successfully fertilised and generated implanting embryos [86]. However the same group also showed that oxygen consumption rates varied depending on the gonadotropin stimulation regime used [86], so care must be taken when comparing oocytes for oxygen consumption and quality. 

While all of these investigations have been informative, they have not led to suitable methods for developing oocytes with good potential or oocyte selection in clinical practice. A noninvasive, reproducible, and high-throughput method for predicting oocyte quality remains to be discovered. If it was possible to do so, one could also investigate ovarian stimulation regimens to find one with the most potential for developing quality oocytes. Studies have promoted the use of lower-dose ovarian stimulation protocols to reduce the risk of aneuploidy in developing oocytes [87]. Other advantages of using milder stimulation are that it is less costly, quicker, and simpler and may be seen as a more natural, “patient-friendly” alternative. This has been recently reviewed [88, 89]; however both reviews concluded that so far there is insufficient evidence to support the use of mild stimulation protocols over standard protocols at this time. Furthermore, a recent study found mild stimulation reduced pregnancy chances without demonstrating cost advantages [90]. A new “freeze all” policy for embryo transfer is also gaining interest, whereby all embryos from a stimulated cycle are vitrified and then the embryo(s) transferred subsequently in a natural cycle [91]. This approach is considered safer than transfer during a stimulated cycle since it may reduce the risk of ovarian hyperstimulation syndrome [91] and the endometrium may be more receptive to embryo implantation compared to when there are a large number of follicles [92]. A large trial (“Elective Vitrification of All embryos (EVA)”) by Genea in Australia is currently being performed to investigate this [93]. It has also been reported previously that vitrifying and transferring embryos in natural cycles give significantly higher pregnancy rates than for stimulated samples [94].

### 3.3. Metabolic Profiling of Follicular Fluid

FF provides the microenvironment for the oocyte in the follicle and is therefore a window into the metabolites available to the oocyte during its development and at the time of ovulation, and also the metabolites excreted by the oocyte and the follicular cells. Therefore it is not unreasonable to postulate that the composition of FF could be indicative of the quality of the oocyte. Furthermore, FF would be an ideal medium in which to test oocyte quality since it is obtained along with the oocyte during follicular aspiration as part of the standard IVF procedure and is usually discarded. However, it must be noted that human oocytes exhibit high levels of aneuploidy, which would not be detected by FF analysis. There have been investigations on the predictive value of specific characteristics of FF and follicles, such as FF volume, follicle diameter, number of follicles [95, 96], but thus far, investigations of FF biomarkers of oocyte quality have mainly been targeted molecular analyses, where a selective class of molecules is profiled [23]. The majority of these studies have been aimed at measuring FF levels of steroid hormones, predominantly progesterone, and oestradiol. Results have been conflicting, with some studies reporting high levels of progesterone or oestradiol to be indicative of good oocyte quality [97–103], some reporting high levels of either hormone to be indicative of poorer quality [101, 104], while other studies have found no correlation between FF progesterone or oestradiol levels and oocyte quality [98, 104–110]. Similarly, prolactin levels have been found to be positively correlated [102, 105], negatively correlated [99, 100], and uncorrelated [106, 108]. Higher levels of FF luteinising hormone (LH) [102] and androstenedione [98] and lower levels of testosterone [100] have been associated with pregnancy; however these findings have been contradicted [105–108]. Other FF hormones such as growth hormone [102], inhibin B [111, 112], leptin [113, 114], angiotensin II [115], cyclic adenosine monophosphate (cAMP) [101, 105, 107], and anti-Mullerian hormone (AMH) [116, 117] have been found to correlate with oocyte potential, whereas follicle stimulating hormone (FSH) has been found to have no correlation [105, 107]. Cytokines have also been measured in FF and correlated to oocyte quality [102, 118, 119], but most of the cytokines studied were not found to have predictive value [110, 120–122]. Other FF peptides and proteins have been investigated as markers of oocyte potential [123–129]. FF levels of apolipoproteins have been found to vary in young and old IVF patients and so could be implicated in age-related infertility [130]. 

In terms of targeted analyses of low molecular weight entities, FF levels of metabolites involved in the homocysteine pathway (vitamins B9, B12, and homocysteine) [131, 132], *myo*-inositol [133], D-aspartic acid [134], alanine, and glycine [135] have been found to have predictive value with regard to IVF outcome. That is to say, the FF levels of these metabolites have been found to correlate with various measures of IVF outcome (oocyte/embryo quality and pregnancy rates) and so measuring their levels in FF may have predictive value on IVF outcome.

The redox state of FF has also been investigated, revealing a significantly elevated oxidised state in FF from oocytes that degenerated compared to those that were normal [136]. Higher levels of reactive oxygen species (ROS) and lower levels of antioxidants have been found in the FF of women who failed to become pregnant following intracytoplasmic sperm injection (ICSI) compared to those who did [137].

Lower FF levels of nitrates and nitrites, produced from granulosa-synthesised nitric oxide (NO), have been correlated with oocyte maturation, fertilisation, embryo development, and implantation in humans [138–140]. Furthermore, a correlation between FF NO concentrations and peri-follicular blood flow (PBF) has been found [141], and a higher PBF is known to be associated with a higher oocyte quality. However, other studies have found no relationship between FF NO and oocyte potential [142] or ovarian response to gonadotrophin stimulation [143].

Targeted studies attempting to identify FF biomarkers of oocyte quality specifically in women with PCOS have also been performed, where leptin [114] and homocysteine [144] have been found to have predictive value and AMH does not [145]. The potential of metabolomics to identify FF markers of oocyte quality specifically in PCOS IVF patients has also been hypothesised [146].

Metabolite profiling studies have been conducted on FF to investigate the effects of diet on oocyte quality. Warzych et al. [147] measured FF levels of fatty acids, using gas chromatography (GC), in pigs fed either a control diet or a diet with elevated fatty acid levels. They found that diet had a significant influence on the fatty acid content of FF and suggested that the alterations in certain fatty acids had a positive impact on oocyte quality [147]. Contradicting this, Sinclair et al. [135] found FF fatty acid composition had no predictive value with regard to oocyte quality in cows. 

The studies mentioned above have provided a wealth of information, but still no single molecule has been identified in FF as a clinically usable biomarker for oocyte quality or pregnancy outcome [23]. Therefore, research into FF biomarkers is also progressing towards metabolomics [23]. Incidentally, an LC-MS-MS proteomics study of human FF has recently been conducted, identifying 246 proteins [148]. Thus, future studies may also be aimed at identifying protein markers of oocyte quality. Bender et al. [149] have recently used GC-MS-based metabolomics to compare the FF of lactating cows and heifers and found significant differences in 24 fatty acids and 9 water-soluble metabolites. The authors discussed the negative effects of a high FF saturated fatty acid content on oocyte maturation and early embryo development, and since the cows had higher levels of these, it was concluded that the FF composition of cows may place their oocytes at a developmental disadvantage, which may contribute to their lower fertility compared to heifers [149]. GC-MS and multivariate statistical methods have also been used to positively correlate FF contamination levels of endocrine-disrupting chemicals (through industrial exposure) with poor fertilisation and oocyte developmental potential, and to predict these FF levels from corresponding blood serum samples [150].

In 2012, Wallace et al. [151] published the first metabolomics study of human FF to assess oocyte viability. Samples collected from patients undergoing stimulated IVF were analysed using ^1^H-NMR spectroscopy, and projections to latent structures by means of partial least squares-discriminant analysis (PLS-DA) were used to discriminate the metabolic profiles based on oocyte embryonic development potential and pregnancy test outcome [151]. Lower FF levels of lactate and choline/phosphocholine and higher levels of glucose and high-density lipoproteins (HDL) were associated with oocytes which failed to cleave as an embryo compared to oocytes producing two-cell embryos (though this PLS-DA model was not validated using an external prediction set) [151]. In terms of pregnancy test outcome, patients who tested positive were associated with a lower FF level of glucose and higher levels of proline, lactate, leucine, and isoleucine [151]. This study successfully demonstrated the potential of ^1^H-NMR-based metabolomics for the analysis of FF from IVF patients and indicated that FF may be predictive of treatment outcome [151]. However the use of patients undergoing twin embryo transfers with a FF sample corresponding to one of the oocytes meant that FF samples could not be linked to singleton pregnancy with confidence, which highlights the preliminary nature of this research and the need for more studies of this kind. Perhaps the biggest drawback to investigating FF as a predictive medium for IVF outcome is the policy of twin embryo transfers, where, unless a multiple pregnancy arises, it cannot be known which embryo prevailed in the singleton pregnancy and so the FF cannot be linked to the outcome. Therefore FF samples need to be obtained from women undergoing eSET, which increases sampling time and reduces cohort sizes dramatically. Furthermore, sampling may be further limited by controlling for women who do not have reproductive diseases which may present unique metabolic profiles that could interfere with any discrimination between profiles from good and bad quality oocytes. Similarly, alterations in metabolism due to drugs may also interfere with the analysis, and thus, women should ideally all be on the same IVF drug regime. It has recently been suggested that the composition of FF is altered by ovarian stimulation [152, 153], therefore it is important to look for biomarkers of oocyte quality in natural FF first in order to understand the natural processes involved in infertility. Natural cycle IVF patients, with no drug regime and single embryo transfers, would be ideal subjects to make preliminary models from. However natural cycle treatments are rare, and therefore it would take a long time to acquire a statistically relevant number of samples.

A further potential limitation of FF is that it often becomes contaminated with flushing medium contained in the dead space of the needle and tubing during the follicular aspiration procedure in IVF or with aspirate of the previous cycle if flushing is not performed. Flushing medium contains many metabolites naturally found in the FF, including glucose and lactate, and so the levels of metabolites in a flush contaminated sample will not be representative of the true composition of the FF. Similarly contamination of aspirate with that from the previous follicle will affect findings. Recently, we have published a pilot ^1^H-NMR-based metabolomic study of FF and blood plasma from modified-natural cycle (the patients were only administered human chorionic gonadotrophin (hCG)) IVF patients undergoing treatment for male factor or unexplained infertility, to investigate differences in the composition of both fluids during the menstrual cycle [154]. Due to the nature of these inclusion criteria, it was only possible to collect samples for ten patients, and therefore the representation of treatment outcomes (2 clinical pregnancies, 8 no pregnancy) was too poor to investigate biomarkers for these outcomes. However, by collecting samples from patients twice, at different phases of their menstrual cycles, it was possible to investigate the FF and plasma for changes with menstrual phase. The use of natural cycle patients allowed the FF and plasma metabolic profiles to be studied without the effects of gonadotropins and reproductive diseases. Therefore, this study demonstrates the power of using samples from natural cycle IVF patients with strict inclusion criteria to ensure confounding factors do not bias or mask any trends of interest and the ease with which contamination by flush media can be accommodated by a spectral subtraction approach [154].

### 3.4. Plasma Composition and Fertility

Since FF is part derived from blood plasma and can exchange metabolites with blood *via* the blood-follicle barrier, the composition of the plasma may have an influence on the quality of the oocyte. Leroy et al. [155] measured several metabolites (glucose, 3-hydroxybutyrate (3HB), lactate, urea, total protein, triglycerides, nonesterified fatty acids (NEFA), total cholesterol, and ions) in both the serum and FF of cows with large and small follicles. Significant correlations between serum and FF were observed for chloride, glucose, 3HB, urea, and total protein at all follicle sizes, and for triglycerides, NEFA, and total cholesterol for large follicles [155]. The authors concluded that metabolic changes in serum will be reflected in the FF and, therefore, could affect oocyte and granulosa cell quality [155]. Blood plasma can be collected relatively noninvasively, compared to FF, and so would be a preferable medium for a test to predict IVF treatment outcome. Furthermore, tests on two different media would provide more confidence than one alone. 

The majority of studies into the effects of blood constituents on oocyte viability and fertility have been in cows. Insulin-like growth factor-1 (IGF-1) has been shown to have a beneficial effect on bovine embryo development *in vitro* [156–158]. Velazquez et al. [159] measured blood levels of IGF-1 and several other blood components (insulin, urea, NEFA, 3HB, and cholesterol) in embryo donor and recipient cows, as part of a multiple ovulation and embryo transfer program. Correlations were found between IGF-1, insulin, and cholesterol levels and embryo viability in donor cows, but all three had very weak predictive power [159]. No correlations were found between the hormone or metabolite levels and pregnancy rate in recipient cows [159]. Other hormones, such as 17-*β* oestradiol, and progesterone have been shown to have predictive value of embryo quality in the cow [160], and embryo quality was enhanced in goats treated with exogenous insulin [161]. 

Kurykin et al. [162] correlated oocyte morphological quality in repeat breeder and early lactation cows to the levels of several blood constituents (plasma total protein, glucose, total cholesterol, high-density lipoprotein cholesterol, urea, albumin levels, and the activities of aspartate aminotransferase and lactate dehydrogenase). The repeat breeder cows produced a greater number of abnormal oocytes than the early lactation cows, and the abnormal oocytes had higher plasma levels of urea and lower levels of albumin in comparison with the normal oocytes of the repeat breeders and the early lactation cows [162]. In support of this, Ferreira et al. [163] found that cows fed a urea diet had decreased oocyte competence compared to controls.

It has been shown that high levels of NEFA in cow plasma, caused by a negative energy balance postpartum, may have a negative impact on fertility through increased levels of NEFA in the FF [164]. *In vitro*, NEFA has been found to reduce the developmental potential of bovine oocytes [165] and to hamper granulosa cell proliferation and steroid production in bovines [166] and humans [167]. Fouladi-Nashta et al. [168] showed that cows fed a diet with rumen inert fat gave a higher oocyte cleavage rate than those fed on diets with soya bean or linseed as the main fatty acid source. However the dietary fatty acid source did not have an effect on development to the blastocyst stage [168]. A previous study by the same group found that increased dietary fatty acids improved oocyte quality [169], whereas other studies [170, 171] found no differences in oocyte quality in cows fed diets with different fatty acid sources. This led Fouladi-Nashta and colleagues to conclude that the level of dietary fat is more important to oocyte quality than the type of fat [168]. Furthermore, this group found that fatty acid composition of the follicular granulosa cells was unaffected by the different dietary fatty acids sources, leading them to hypothesise that the ovary (in the cow at least) moderates the uptake of individual plasma fatty acids so as to keep the fatty acid profile to the oocyte constant [168].

In humans, serum AMH has been found to correlate with oocyte and embryo quality in IVF patients [172–177]. Lee et al. [175] showed that in breast cancer patients undergoing oocyte cryopreservation, the likelihood of obtaining more than four mature oocytes was higher for AMH levels >1.2 ng mL^−1^. In another study [174], ICSI patients with AMH levels <1.66 or >4.52 ng mL^−1^ gave oocytes of poorer quality, but AMH was not predictive of fertilisation rate or embryo quality. Elgindy et al. [177] found AMH levels to have the best prognostic value for clinical pregnancy during the midluteal and early follicular phases of ICSI patients. Furthermore, AMH has recently been shown to be predictive of the number of transferable embryos in cows [178] and goats [179]. FSH has also been investigated as a potential predictor of IVF outcome, with some success [180], although other studies found AMH to be a superior predictor [173–175].

Plasma levels of apolipoproteins have been found to vary in young and old IVF patients and so could be implicated in age-related infertility [130]. Apolipoprotein B levels were higher in younger patients than old, though this difference was not statistically significant, apolipoprotein E levels were greater in older patients for low density lipoprotein (LDL) and very low density lipoprotein (VLDL) complexes and apolipoprotein A1 did not differ with age [130]. Protein and peptide levels in serum and FF may also be indicative of follicle maturity and hence oocyte quality [181]. Higher serum levels of soluble triggering receptor expressed on myeloid cells-1 (sTREM-1), which is a marker of infection and inflammation, have been associated with poorer quality embryos [182]. In terms of lower molecular weight metabolites, high plasma levels of vitamin B12 have been associated with better embryo quality [131].

So far, there has not been a global metabolomic study of blood plasma with view to identifying biomarkers of oocyte quality or IVF outcome. While the targeted analyses mentioned above have revealed some potential predictive markers, a metabolomics investigation would allow simultaneous measurement of a greater number of metabolites, many of which have not previously been investigated in IVF patients. 

Metabolomics has however been used to compare plasma from PCOS sufferers and controls. Sun et al. [183] were the first to do this. Using NMR spectroscopy and supervised multivariate statistical analysis, the authors identified significant decreases in plasma amino acids, citrate, choline, and glycerophosphocholine/phosphocholine, and increases in the levels of dimethylamine (DMA), lactate, and *N*-acetyl glycoproteins in PCOS patients compared to controls. Sun et al. [183] interpreted these trends as reflecting the perturbations in amino acid metabolism, impairment of the tricarboxylic acid (TCA) cycle (citrate), increased activity of the gut microflora (citrate, choline and glycerophosphocholine/phosphocholine) (which has been linked to diabetes, obesity, and cardiovascular disease), increased glycolytic activity (lactate and glucose (insignificant)), and inflammation (glycoprotein). Furthermore, greater metabolic deviations were observed in subgroups of patients with obesity, metabolic syndrome, and hyperandrogenism [183]. Similarly, Atiomo and Daykin [184] performed NMR-based metabolomics on PCOS and healthy control women shortly after Sun et al. [183], finding significant decreases in citrulline, lipids, arginine, lysine, ornithine, proline, glutamate, acetone, citrate, and histidine in PCOS compared with controls. These studies have demonstrated the potential of NMR-based metabolomics for the study of PCOS, not only for identifying potential biomarkers but also for uncovering associated changes in metabolic pathways, leading to a better understanding of the causes and pathogenesis of PCOS, and offering the potential to find better treatments. No metabolomics studies of blood plasma or any other reproductive fluid or tissue have been performed for other reproductive diseases such as pelvic inflammatory disease (PID) or endometriosis. The diagnosis of PID is very difficult because the disease presents itself in a variety of ways and is still ill defined [185]. Often women presenting with tubal factor infertility have had a past episode of PID, which went undiagnosed due to mild symptoms [186]. PID is currently diagnosed according to a definition proposed by Hager et al. [187], using a mixture of clinical symptoms (such as abdominal pain, vaginal discharge, menstrual irregularities, and urinary symptoms, to name a few), microbial and histological tests, and confirmation with laparoscopy [185]. However, in a critical review of PID diagnosis, Simms et al. [188] concluded that there is insufficient evidence to support existing diagnostic guidelines, and a new evidence base or new diagnostic techniques are required. Furthermore, microbial diagnosis and laparoscopy are both invasive, and laparoscopy is expensive and operator subjective [185]. Therefore a single noninvasive test, such as a blood sample test, to confidently diagnose PID early on would be highly desirable. Endometriosis is also difficult to diagnose because the symptoms are very similar to those of other chronic pain disorders, such as irritable bowel syndrome, and can go undiagnosed for long periods of time [189]. In most cases, surgery is required for a definitive diagnosis [190], which is invasive and leads to long delays before diagnosis [191]. Furthermore, endometriosis can present itself in a number of stages, but the severity of the disease is poorly correlated to the severity of the symptoms [192]. Therefore a noninvasive blood test to predict endometriosis and the severity of the disease would be ideal. PID and endometriosis have very similar symptoms and are often confused, so a diagnostic test that is able to distinguish between these diseases would also be of great clinical value. Metabolomics has the potential to identify such biomarkers in women with these diseases, but as of yet only targeted biomarker investigations have been performed and no biomarkers are in current clinical use.

The potential of blood plasma as a medium for predicting oocyte quality is less great than for FF or culture medium because it is not in direct contact with the oocyte. Furthermore, blood plasma is subject to more variation due to other factors that influence the metabolism (such as diet, level of activity, and disease) than FF or culture medium, which are compartmentalised or isolated from the body, respectively. However, plasma composition may be informative of a general status of a patient, and thus patients with extreme deviations from “normality”, for example, may be associated with a poorer chance of becoming pregnant. Therefore tests on plasma may be of some use in predicting IVF outcome, but are unlikely to be the only test required. Performing a blood test in conjunction with a test on FF, for example, may validate and support a prediction from the FF. Furthermore, *correlations* between FF and plasma may be of diagnostic value. These are potential applications for metabolomics, which are yet to be addressed. 

## 4. Concluding Remarks

Research into biomarkers of IVF outcome is essential if we are to increase the numbers of eSET performed and hence reduce the multiple pregnancy rate without compromising the overall success rate in assisted conception. A large amount of knowledge has been gained over the years from targeted studies of embryo culture medium, oocytes, FF, and plasma, but still no biomarker is in routine clinical use. The move from these studies to metabolomics in recent years has offered a much greater potential for unravelling the mechanisms of infertility and for identify groups of diagnostic markers or models from which new samples can be predicted. However, this research is still in its infancy and the limitations discussed in this paper must be overcome before the desired goals can be achieved. In the pursuit of embryo markers in embryo culture medium, tests that are generally applicable and can cope with different types of medium and procedures used in different clinics are required. Though the majority of metabolomics studies in the field of assisted conception have been focused on embryo selection through the analysis of embryo culture medium, it has so far not proved clinically useful. In oocyte research, methods that are nondestructive to the oocyte are required and FF metabolomics studies should allow the necessary time to collect a good number of representative samples from patients undergoing eSET. Flush contamination should also be accounted for. It must also be considered that metabolism is a dynamic process and therefore sample collection over a time period may prove beneficial in establishing biomarkers through metabolomics analysis. Indeed dynamic modelling studies have been developed in the metabolomics community [193, 194]. However, the primarily goal with metabolomics is usually biomarker identification, not discovering the mechanism of metabolism. Ultimately, if a biomarker can distinguish between two (or more) groups of samples then it is a legitimate biomarker. With such improvements and extensions over the coming years, the possibility of identifying biomarkers of IVF outcome using metabolomics could become a reality, which is a very exciting prospect.
